# The effect of saturated and unsaturated fatty acids on the production of outer membrane vesicles from *Bacteroides fragilis* and *Bacteroides thetaiotaomicron*

**Published:** 2019

**Authors:** Zahra Sadat Mirjafari Tafti, Arfa Moshiri, Fateme Ettehad Marvasti, Samira Tarashi, Seyedeh Fatemeh Sadati Khalili, Atiyyeh Motahhary, Abolfazl Fateh, Farzam Vaziri, Sara Ahmadi Badi, Seyed Davar Siadat

**Affiliations:** 1 *Microbiology Research Center, Pasteur Institute of Iran, Tehran, Iran*; 2 *Gastrointestinal Cancer Department, Gastroenterology and Liver Diseases Research Center, Research Institute for Gastroenterology and Liver Diseases, Shahid Beheshti University of Medical Sciences, Tehran, Iran*; 3 *Experimental Therapy Unit, Laboratory of Oncology, G.Gaslini Children’s Hospital, Genoa, Italy*; 4 *Mycobacteriology and Pulmonary Research Department, Pasteur Institute of Iran, Tehran, Iran *

**Keywords:** Bacteroides fragilis, Bacteroides thetaiotaomicron, Outer membrane vesicle, Palmitic acid, Palmitoleic acid

## Abstract

**Aim:**

The aim of present study is to investigate the effect of fatty acids on the outer membrane vesicles (OMVs) produced by *Bacteroides* spp.

**Background:**

*Bacteroides *spp. is the important member of Gut microbiota that employ OMVs production for interact with host. Besides, dietary fatty acids could influence on determination of gut microbiota composition and immune response. In this regard, we evaluated the effect of fatty acids on the growth and OMVs production of *Bacteroides fragilis* and *Bacteroides thetaiotaomicron*.

**Methods:**

*B. fragilis *and* B. thetaiotaomicron *were grown on BHI broth with and without palmitic and palmitoleic acids as saturated and unsaturated fatty acids, respectively. OMVs were extracted using multiple centrifugation and tris-ethylene diamine tetra acetic acid (EDTA)-Sodium deoxy cholate buffers. Physicochemical properties of OMVs were detected by electron microscopy (SEM), Bradford Coomassie brilliant blue assay and SDS-PAGE. Data were analyzed with One-way ANOVA using SPSS.

**Results:**

The growths of both *Bacteroides* were significantly increased by palmitic acid. Nevertheless, palmitoleic acid had no significant effect on them. Palmitic acid significantly decreased and increased the production of *B. fragilis *OMVs at low and high concentration, respectively. However, the production of *B. thetaiotaomicron* OMVs was not significantly affected by palmitic acid. Although palmitoleic acid had a significant decreasing effect on the production of *B. fragilis* OMVs, it significantly increased the production of *B. thetaiotaomicron* OMVs at low concentration.

**Conclusion:**

In conclusion we reported that palmitic acid had a stimulatory effect on the growth of *B. fragilis *and* B. thetaiotaomicron* and had a dose dependent effect on the production of *B. fragilis* OMVs. Also producing of *B. thetaiotaomicron *OMVs was affected by palmitoleic acid in a dose dependent manner.

## Introduction

 A diverse and complex microbial community colonizes human host, especially in the gastrointestinal tract (GIT) that refers as the “gut microbiota”. This microbial community has co-evolved with host to create a mutually beneficial relationship (1). Gut microbiota constantly interacts with the host and has determining roles in its functions due to having various physiological effects including maintenance of intestinal epithelium integrity, energy harvest from diet, colonization resistance, regulation of glucose/lipid metabolism and immune system (2, 3, 4). The gut microbiota consists of bacteria, archaea, protozoa, fungi and viruses. The *Bacteroidetes* and *Firmicutes* are two dominant phyla in human gut microbiota (5).The composition of the gut microbiota is affected by genetic background and environmental factors including toxins, drugs, diet and pathogens (6). It has been found that unfavorable altered gut microbiota composition that is called “dysbiosis”, disrupts gut microbiota-host interactions which increases prone to diseases. Dysbiosis is correlated with intestinal and extra-intestinal disorders like irritable bowel syndrome (IBS), coeliac disease, inflammatory bowel disease (IBD), asthma, allergy, cardiovascular disease (CVD), metabolic syndrome and obesity (6-10). It has been suggested that diet is one of the most potent determinants of gut microbiota composition. It affects gut microbiota-host interactions through alternation of microbial metabolites, components and host metabolism. For example, dietary fatty acids have influential role on metabolic syndrome such as obesity, type 2 diabetes, hyper tension and rheumatoid arthritis (11-14). Dietary saturated fatty acids (SFAs) such as palmitic acid are able to activate inflammatory responses and promote metabolic syndrome. Conversely, polyunsaturated fatty acids (PUSAs) could suppress inflammatory responses (15). 

The gut microbiota has significant roles on human physiology and metabolism. It produces essential metabolites from diet such as short-chain fatty acids (SCFAs) which act as source of energy for colonocytes, signaling molecules and epigenetic factor for modulation host functions (16, 17). Also immune system and host’s defenses are associated with composition and function of microbiota (16-18). One way to interact between gut microbiota and the host is to produce outer membrane vesicles (OMVs). OMVs are nano sized particles, 20 to 250 nm, which produced by gram negative bacteria (19, 20). The component of OMVs includes proteins, hydrolytic enzymes, toxin or lipopolysaccharide (LPS), DNA and RNA (21, 22). Recent studies demonstrated that *Bacteroides* spp. derived OMVs have significant role in maintenance of homeostasis and regulation of immune system. For example, OMVs containing capsular polysaccharide A (PSA) from *B. fragilis* modulate the immune system and tolerance to intestinal commensal bacteria. *B. thetaiotaomicron* OMVs modulate intestinal macrophages in a sulphatase dependent manner. Also hydrolytic enzymes that are packaged into *Bacteroides* spp. derived OMVs, contribute to maintenance of homeostasis (19-27). As mention above, the gut microbiota composition is affected by many factor especially diet, dietary SFAs and PUSAs. According to importance of dietary fatty acids and *Bacteroides* spp. and their OMVs, we evaluated the effect of palmitic and palmitoleic acids, as saturated and unsaturated fatty acids, on the growth and the production of OMVs from *B. fragilis* and *B. thetaiotaomicron*. 

## Methods

**Bacterial strains and growth condition**

*B. fragilis *ATCC 23745 and *B. thetaiotaomicron *ATCC 10774 were grown in brain heart infusion (BHI) broth supplemented with hemin (5µg/ml) and menadione (1µg/ml). These media incubated at 37 °C under anaerobic conditions provided 80% N_2_, 10% Co_2_ and 10% H_2_.

**Fatty acids preparation and bacterial inoculation**

Palmitic acid (PO500) and palmitoleic acid SIGMA-ALDRICH, GERMANY (P9417) solutions were prepared at 20 mg/ml concentration in absolute ethanol. Fatty acid solutions were sterilized using a 0.22µm-pore-size polyvinylidene difluoride filter (Millipore, Billerica, MA). Palmitic acid and palmitoleic acid solutions were added to BHI broth media at different concentration, 0, 125, 250 and 500 µg/L. *B. fragilis *and *B. thetaiotaomicron* were inoculated at 1.5×10^8^ CFU ml^-1^ to BHI broth enriched with palmitic and palmitoleic acid and incubated under anaerobic conditions for an overnight. Finally, the optical density (OD) was measured by ELISA reader (Epoch Biotech ELX50)*. *

**OMVs purification**

After an overnight incubation under anaerobic conditions as described previously, OMVs were isolated (28). Briefly, five hundred milliliters of bacterial cultures were centrifuged at 5000g at 4 °C. The cell pellets were washed twice by phosphate buffer solution (PBS). Then cell pellet was re-suspend in sodium chloride 9% solution. The cell suspension was homogenized and concentrated by centrifugation for 1 hour at 6500g at 4 °C. The OMVs were isolated using Tris-ethylene diamine tetra acetic acid (EDTA)-sodium deoxycholate (Sigma-Aldrich, USA) buffers and centrifugation for 90 minutes at 20000g, 4 °C. The OMVs concentrated were re-suspended in 3% sucrose solution.

**Scanning electron microscopy (SEM)**

To determine morphology and size of OMVs, these vesicles were fixed using 2.5% glutaraldehyde and 2% paraformaldehyde in PBS. Gold coated samples were prepared by sputter coater (KYKY Technology, China) and examined using SEM (KYKY Technology, China). 

**Figure 1:**
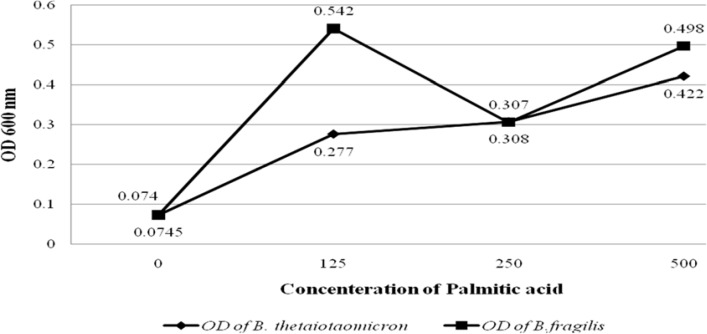
Graph shows the OD values of *B. fragilis *ATCC23745 and *B. taiotaomicron *ATCC 10774 growths in BHI broth treated with palmitic acid. The horizontal and vertical axes show the concentration of Palmitoleic acid and the OD values of bacterial growth, respectively

**Figure 2:**
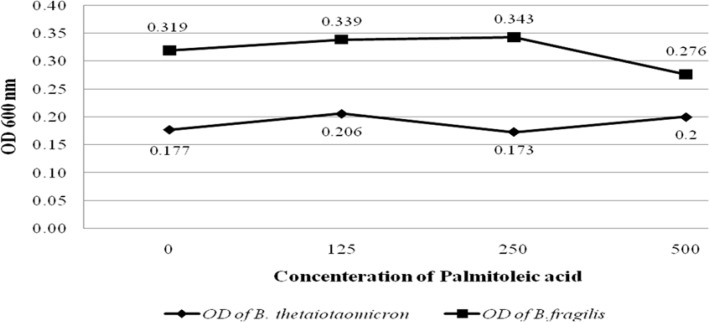
Graph shows the OD values of *B. fragilis *ATCC23745 and *B. thetaiotaomicron *ATCC 10774 growths in BHI broth treated with Palmitoleic acid. The horizontal and vertical axes show the concentration of Palmitoleic acid and the OD values of bacterial growth, respectively

**Figure 3:**
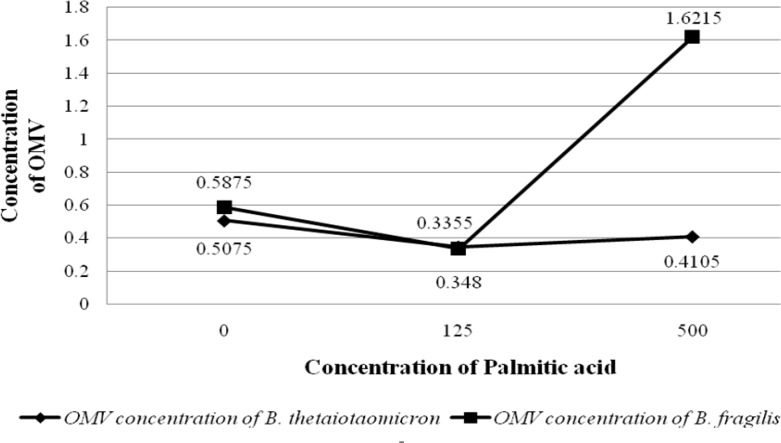
Graph shows the OD values of OMVs production by *B. fragilis *ATCC23745 and *B. thetaiotaomicron *ATCC 10774 in BHI broth supplemented with Palmitic acid. The horizontal and vertical axes show the concentration of Palmitoleic acid and the OD values of bacterial growth, respectively

**Figure 4:**
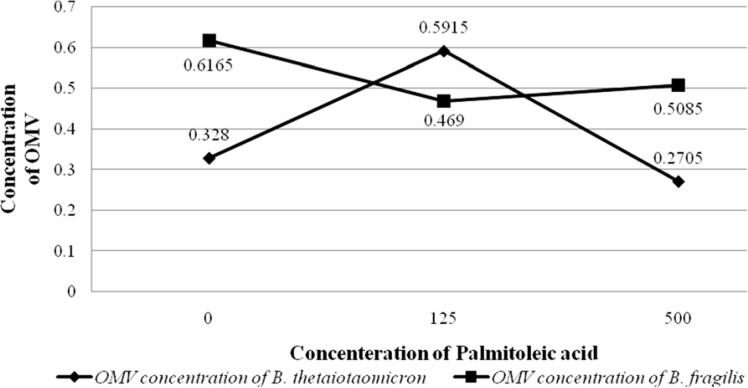
Graph shows the OD values of OMVs production by *B. fragilis *ATCC23745 and *B. thetaiotaomicron *ATCC 10774 in BHI broth supplemented with Palmitoleic acid. The horizontal and vertical axes show the concentration of Palmitoleic acid and the OD values of bacterial growth, respectively

**Figure 5:**
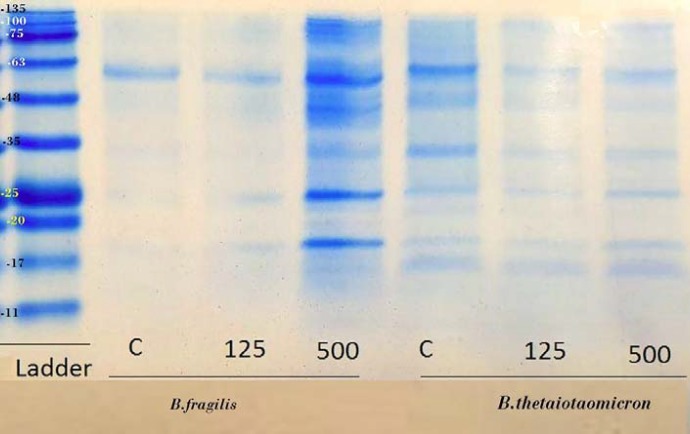
The protein profile of *B. fragilis *ATCC23745 and *B. thetaiotaomicron *ATCC 10774 derived OMVs which are produced in BHI broth supplemented with high and low concentration of palmitic acid. The protein profiles of OMVs from *B. fragilis *and *B. thetaiotaomicron *were compared using SDS-PAGE according to Claassen et al. (1996)

**Figure 6:**
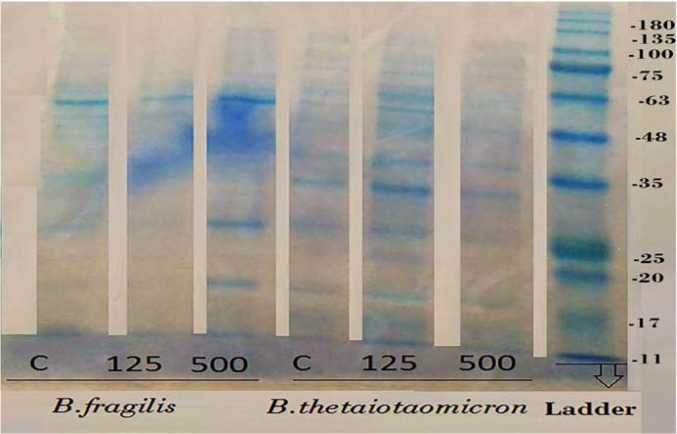
The protein profile of *B. fragilis* ATCC23745 and *B. thetaiotaomicron* ATCC 10774 derived OMVs which are produced in BHI broth supplemented with high and low concentration of palmitoleic acid. The protein profiles of OMVs from *B. fragilis* and *B. thetaiotaomicron* were compared using SDS-PAGE according to Claassen et al. (1996)

**Quantitative and qualitative determination of protein**

 After the extraction, the protein concentration of OMVs was measured by Bradford protein assay. Determination of OMVs proteins by Bradford Coomassie brilliant blue assay was confirmed by measuring absorbance at 590 nm (29). To determine protein profile electrophoresis of OMV was performed by slab gel containing 12% SDS-PAGE. Then separated proteins were stained with Coomassie brilliant blue and were de-stained by acid acetic 1 %( 30).

**Statistical Analyses**

Data were analyzed with One-Way ANOVA based on LSD test using SPSS version 24.0 (SPSS Inc., Chicago, IL, USA). All results demonstrate as mean± Standard deviation (SD). In all experiments, P<0.05 was considered statistically significant. 

## Results

**The effect of palmitic and palmitoleic acids on** ***B. fragilis*** **and** ***B. thetaiotaomicron*** **growth**

To evaluate the effect of palmitic and palmitoleic acid on the growth of the *B. fragilis *and *B. thetaiotaomicron, *different concentration of both fatty acids was added to BHI broth. The results showed that palmitic acid significantly increased the growth of *B. fragilis *(*P value* < 0.05) and* B. thetaiotaomicron* (*P value* < 0.05). Palmitic acid had most stimulatory effect on these bacteria at the high concentration, 500µg/L. Interestingly, the growth of *B. fragilis* was more stimulated compared with *B. thetaiotaomicron* by palmitic acid (figure 1). In contrast, palmitoleic acid had no significant stimulatory effect on the growth of the *B. fragilis* and* B. thetaiotaomicron *(figure 2). 

**The effect of palmitic acid and palmitoleic acid on the production of** ***B. fragilis*** **and** ***B. thetaiotaomicron*** **derived-OMVs**

To evaluate the effect of palmitic acid and palmitoleic acid on the OMVs production of *B. fragilis *and* B. thetaiotaomicron*, these particles were extracted in BHI broth supplemented with different concentrations of fatty acids examined by SDS and Bradford methods that results of the experiments are shown in the figures (figure 3 and 4). Although palmitic acid significantly decreased the OMVs production of *B. fragilis *at low concentration (*P value* < 0.01) but significantly increased it at high concentration (*P value* < 0.002). Based on statistical analyzes; it was showed that palmitic acid effect on the production of OMVs from* B. fragilis* was dose dependent due to the presence of a significant increasing effect at high concentration (*P value* < 0.001). The production of *B. thetaiotaomicron *OMVs was not significantly affected by palmitic acid at both concentrations (figure 3 and 4). On other hand, palmitoleic acid had a decreasing effect on the production of *B. fragilis* OMVs and the low concentration was statistically significant (*P value* < 0.03). We reported that the palmitoleic acid had a significant increasing effect on the production of *B. thetaiotaomicron *OMVs at low concentration (*P value* < 0.03) (Figure 5 and 6). Also, we identified a dose dependent effect of palmitoleic acid on the *B. thetaiotaomicron *OMVs production because of the high concentration compared with low concentration had a significant reducing effect (*P value* < 0.01) in this regard.

## Discussion

*Bacteroides* spp. have significant roles in the gut microbiota-host interactions through various mechanisms including OMVs production. These particles influence the regulation of immune system and homeostasis (31, 32). On the ther hand, diet-derived saturated and unsaturated fatty acids have inflammatory and anti-inflammatory properties, respectively (33, 34). Here, we aimed to evaluate the effects of palmitic and palmitoleic acids (as saturated and unsaturated fatty acids) on the growth and the production of OMVs from *B. fragilis* and *B. thetaiotaomicron*.

It has been shown that diet has substantial role on the gut microbiota composition (35). In this regard, some studies focused on the role of high fat diet as an important factor on the pattern of gut microbiota (36, 37). Also, the effect of dietary fatty acids on the intestinal bacterial growth was assessed (38). For example, fatty acids derived from human and cow milk inhibits the growth of *Lactobacillus bifidus* (39). Furthermore, the growth of *Bacteroides ruminicola*, *one* species of rumen bacteria, was decreased in the presence of palmitic acid (40). Some researches indicated that unsaturated long-chain fatty acids (C18) have stimulation effect on the growth of microorganisms in low concentration (41). In present study, palmitic acid acts as a significant stimulant for the growth of both *B. fragilis *and* B. thetaiotaomicron*, while palmitoleic acid has no significant effect on the growth of these bacteria. The stimulatory effect of unsaturated fatty acids on the growth of bacteria decreases along with the increase in the length of the fatty acid chain. Also, fatty acids which have induction effect on the bacterial growth might have opposite effect at other concentration. Therefore, fatty acids can be considered as a stimulator or inhibitor agent on the bacterial growth depending on their structure and concentration (41).

*B. fragilis *and* B. thetaiotaomicron* are two important members of gut microbiota due to having important potentials such as regulating of immune responses and homeostasis (42, 43). It has been demonstrated that, *B. fragilis *and* B. thetaiotaomicron *employ OMVs production to exert their effects in gut microbiota-host interactions (44, 45). Considering the important roles of *Bacteroides* spp. derived OMVs, the effects of palmitic acid and palmitoleic acid which are components of diet, on the production of these vesicles were investigated for the first time. Our results illustrate that palmitic acid has a significant stimulatory effect on the production of *B. fragilis *derived OMVs at the high concentration but it significantly decreased production of *B. fragilis *OMVs at low concentration. However, palmitic acid had no significant effect on the production of *B. thetaiotaomicron *OMVs. Palmitoleic acid, as an unsaturated fatty acid, significantly decreased the OMVs production from *B. fragilis *and significantly increased the production of *B. thetaiotaomicron* OMVs at low concentration. It can be concluded that the effect of palmitic acid on the production of OMVs from* B. fragilis* and also the effect of palmitoleic acid on the production of OMVs from *B. thetaiotaomicron *was dose dependent. Therefore, fatty acids can act as a stimulator or inhibitor agent on the production of OMVs depending on their concentration. However, further molecular investigations and animal models are required to illustrate in details the effect of palmitic and palmitoleic acid on the growth of *Bacteroides* spp. and OMVs production.
